# GRIEF LITERACY: COMMUNITY-BASED STRATEGIES TO REFRAME GRIEF IN OLDER ADULTS

**DOI:** 10.1093/geroni/igae098.3344

**Published:** 2024-12-31

**Authors:** Katherine Supiano, Bradbury Laura

**Affiliations:** University of Utah, Salt Lake City, Utah, United States; University of Utah, Salt Lake City, Utah, United States

## Abstract

Grief Literacy is the ability to be compassionately present with a person who is suffering. Older adults realize more grief experiences than the population in general, and have the highest likelihood of poor bereavement outcomes, including risk for Prolonged Grief Disorder. Recognizing that our society is fragmented, and the varying norms and traditions to support one another in times of suffering are unclear, increasing grief knowledge would equip both the public and professionals to identify grief and suffering more readily and to activate appropriate supports to be proactive in avoiding complications from the grief and suffering such as isolation and suicide-risk. In addition to formal care offered by palliative and hospice care professionals, grief counselors, and mental health clinicians, the Grief Literacy Movement equips all citizens to support one another in times of loss, suffering and bereavement. We developed and implemented a year-long State-funded program to promote grief literacy across the State of Utah, targeting persons most impacted by the pandemic; older adults, indigenous persons, Veterans, rural communities—and their overlapping constituencies. Rather than confining this effort to provision of support, we developed grassroots strategies for mutual empowerment of community members to reframe and address individual and collective loss. We present applications including education of mental health providers/health providers, first responders, religious communities, and the engagement of citizen groups. We will provide outcomes of our framework applied across multiple social and community settings that enhance supportive care and contribute to mitigating risk of interpersonal harms that can affect bereaved older persons.

